# Critical pre- and postoperative factors in evaluating ptosis

**DOI:** 10.3389/fopht.2025.1609113

**Published:** 2025-08-04

**Authors:** Jordan N. Cornwell, Claire S. Smith, Katherine M. Lucarelli, Alexander Sones, Daniel B. Rootman

**Affiliations:** Division of Orbital and Ophthalmic Plastic Surgery, Doheny and Stein Eye Institutes, University of California, Los Angeles, Los Angeles, CA, United States

**Keywords:** ptosis repair, pre-operative assessment, ptosis-related outcomes, delphi technique, nominal group technique

## Abstract

**Background:**

Blepharoptosis repair is a common eyelid surgery worldwide, however technique and outcome measurements vary widely.

**Objective:**

This study aims to determine a consensus on pre- and postoperative factors important to oculoplastic surgeons in the evaluation of ptosis.

**Methods:**

Oculoplastic surgeons were queried to describe 10 or more variables important for pre- and postoperative evaluation of ptosis and subsequently rate them on a 5-point Likert scale. A nominal group meeting determined a consensus on the 10 most important pre- and postoperative factors based on the previous survey responses. Mean and standard deviation for Likert score responses were compared using a student’s t-test.

**Results:**

One hundred and sixty-three respondents contributed a total of 1,909 open-ended responses. The two most cited factors were levator function (91.4%) and upper marginal reflex distance (87.7%). Known secondary causes of ptosis (75.5%), health of cornea and tear film (58.9%) and brow elevation/compensation (55.2%) were reported as important by over 50% of the respondents. The final variables were categorized into four groups: Causes of Ptosis, Surgical Planning, Anatomy and Symmetry, and Ptosis-Related Quality of Life.

**Conclusion:**

A cohort of ASOPRS oculoplastic surgeons reached a consensus on a critical set of variables important for ptosis evaluation.

## Introduction

Blepharoptosis is one of the most common eyelid malpositions, with an estimated prevalence of over 10% in adult populations (1). This broad diagnosis encompasses a wide range of etiologies, though the vast majority would be classified as involutional. Blepharoptosis is commonly surgically corrected, with more than 86,000 procedures occurring per year and an approximately $46 million-dollar annual associated cost in the United States in 2023 (1, 2).

Despite the relative frequency of this malposition and its treatment, outcomes may be unpredictable, with re-operation rates ranging from 5%-35% depending on etiology and technique (2–9). Given the variability in outcomes, it is important to have a consensus on pre- and post-operative evaluation metrics. A universal definition of outcome success is highly variable in the literature. Outcome measures reported include margin reflex distance one (MRD1), amount of ptosis (defined as difference from a normal upper eyelid height of 4 or 4.5 mm), degree of ptosis (defined as MRD1 <2 mm, <2.5 mm, or <3 mm), acceptable eyelid contour (defined subjectively or objectively), symmetry between the eyelids, tarsal platform show, reoperation rates, and complication rates (11–14).

With individual surgeons relying on different measures to determine candidacy for surgery and to define successful outcomes (3, 6, 10, 11), standardization and comparison of ptosis surgery techniques in the literature can be challenging. The purpose of this investigation is to determine a consensus from the surgeon’s perspective on important pre- and post-operative metrics for the evaluation of ptosis.

## Methods

This study complied with the University of California Los Angeles Institutional Review Board policies (IRB #15-000949) and principles and was conducted in accordance with the Declaration of Helsinki.

In this qualitative study, ptosis surgery experts were surveyed for participation. The experiment involved two stages. The first utilizing a survey-based Delphi exercise to generate a comprehensive list of ranked factors in assessing ptosis. The second stage involved a nominal group exercise intended to group, prioritize and organize these factors into a parsimonious consensus set of variables.

The Delphi technique is a consensus-building process in which panels endorse or reject opinions or assumptions based on an iterative series of questionnaires with controlled feedback (15). Before each round of inquiry, participants are presented with a summary of other members’ responses and encouraged to reconsider their answers until consensus is achieved (16, 17). The Delphi technique concludes with a group ranking of the importance of the comprehensive set of variables of interest.

The nominal group technique is a method of reaching consensus from a panel of experts (18, 19). This exercise involves presenting a series of standardized questions to the panel and obtaining uninterrupted oral responses to every question from each participant. Open ended discussion follows each round of responses as well as the conclusion of the standardized questions. In the present study, the nominal group technique was used after the Delphi exercise to refine the outcome measures of interest previously identified.

### Delphi exercise

Members of the American Society of Ophthalmic Plastic and Reconstructive Surgery (ASOPRS) were solicited via email for participation in the Delphi technique. The open-ended prompt asked respondents to identify 10 or more variables used to evaluate a patient with ptosis in both the preoperative and postoperative state. The responses were collated into semantically equivalent groups and sorted by the percentage of respondents identifying each variable. The same cohort of survey responders was then re-queried in a secondary survey to rate the relative importance of each variable in evaluating a person with ptosis pre- or postoperatively. Answers were graded on a Likert scale from 1-5, with 1 being least important and 5 being most important. Variables identified in the Delphi exercise with an average score of above 3.0 on the 5-point Likert scale were considered to be important and included in the nominal group round.

### Nominal group

The nominal group was additionally composed of ASOPRS oculoplastic surgeons. The group meeting involved first a silent generation of variables. Variables identified through the Delphi technique with Likert scores below 3.0 were then silently reviewed for re-inclusion. Newly identified variables, variables from the Delphi analysis scoring 3.0 and above, and variables scoring below 3.0 that were re-evaluated and included by the group were then discussed in round robin format without interruption. The group discussion of each variable continued until consensus was achieved on the 10 most important variables in evaluating ptosis in the pre- and postoperative periods respectively.

The nominal group focused on the inclusion of reliable, feasible, measurable, and critical variables and the elimination of redundant variables. The discarded variables were re-reviewed for inclusion and the final list of variables was determined by group consensus. The final variables were grouped and categorized based on their importance to different aspects of the surgical process.

### Statistical analyses

Mean and standard deviation for Likert score responses for each variable were calculated. Mean Likert scores for the preoperative and postoperative importance of variables were evaluated for significant differences utilizing the student’s t-test.

## Results

### Delphi

In the open-ended survey, 163 respondents contributed a total of 1,909 responses. After collation, a total of 59 unique factors were identified. The two most commonly cited factors were levator function (91.4%) and upper margin reflex distance (MRD1) (87.7%). Known secondary causes of ptosis (75.5%), health of cornea and tear film (58.9%) and brow elevation/compensation (55.2%) were also reported as important variables by over 50% of the respondents. All other variables were noted by fewer than half of respondents.

Seventy-three of the 163 respondents participated in the second round of Delphi Analysis, ranking the previously identified factors on a 5-point Likert scale. Mean and standard deviation of Likert scale rankings were calculated and compared to determine statistically significant differences in the pre- and postoperative means for each variable (**Tables 1**–**3**).

**Table 1:** Factors with Likert scores >3.5 in which preoperative grading of importance was significantly higher than postoperative.

Variable	% Respondents describing	Mean Pre-operative Likert (SD)	Mean Post-operative Likert (SD)	p-value
Levator function	91.4%	4.9 (0.32)	3.2 (1.41)	<0.01
Known secondary cause of ptosis	75.5%	4.3 (0.82)	3.1 (1.15)	<0.01
Ocular motility	47.9%	3.6 (0.99)	3.1 (1.06)	<0.01
Congenital vs acquired	37.4%	4.2 (0.83)	2.7 (1.32)	<0.01
Eyelid surgery or trauma	31.9%	3.6 (0.95)	2.6 (1.05)	<0.01
Orbicularis / CN VII function	28.2%	3.6 (1.10)	3.1 (1.21)	<0.05
Herring's positivity	22.7%	3.6 (1.00)	3.1 (1.17)	<0.05
Anticoagulation	9.2%	3.7 (1.30)	1.7 (1.00)	<0.01
Proptosis/orbital asymmetry	8.0%	3.6 (1.06)	2.6 (1.29)	<0.01
Prior history of ptosis surgery	4.9%	4.0 (0.98)	2.5 (1.35)	<0.01

**Table 2:** Factors with Likert scores >3.5 in which postoperative grading of importance was significantly higher than preoperative.

Variable	% respondents describing	Mean Pre- operative (SD)	Mean Post- operative (SD)	p-value
MRD1	87.7%	4.4 (0.90)	4.7 (0.65)	<0.05
Lid crease symmetry	4.3%	3.3 (1.12)	3.8 (1.17)	<0.01
Lid contour	6.1%	3.4 (1.02)	3.9 (1.11)	<0.05
Symmetry of eyelid position	22.1%	3.5 (1.10)	4.1 (1.09)	<0.01

**Table 3:** Factors with Likert scores >3.5 in which preoperative and postoperative grading of importance were not significantly different.

Variable	% respondents describing	Mean Pre- operative (SD)	Mean Post- operative (SD)	p-value
Health of cornea and tear film	58.9%	4.1 (0.93)	4.3 (0.822)	0.069
Brow elevation/compensation	55.2%	3.7 (1.00)	3.5 (1.00)	0.161
Lid crease height/position	46.0%	3.4 (1.02)	3.6 (1.05)	0.333
Dermatochalasis	42.9%	3.5 (0.96)	3.3 (0.95)	0.414
Lagophthalmos	36.2%	4.0 (1.00)	4.2 (1.00)	0.363
Dry eye	35.6%	3.8 (1.07)	4.1 (0.93)	0.097
Expectations for surgical outcome	25.2%	3.9 (1.08)	4.0 (1.00)	0.577
Lid lag/eyelid retraction	22.1%	3.5 (1.17)	3.4 (1.24)	0.907
Ptosis-related quality of life	20.2%	3.3 (1.29)	3.5 (1.27)	0.473
Vertical palpebral height	18.4%	3.2 (1.28)	3.5 (1.24)	0.203

Factors with significantly higher Likert scores in the preoperative assessment relative to the postoperative assessment included ocular motility, previous eyelid surgery or trauma, facial nerve function, orbital asymmetry, congenital nature of disease, and Hering’s positivity (**Table 1**). Factors with Likert scores that were significantly higher in the postoperative assessment relative to the preoperative assessment included MRD1, lid crease symmetry, contour, and overall symmetry (**Table 2**). A range of variables were found to be equally important in the preoperative and postoperative assessment, including lagophthalmos, dry eye, eyelid retraction, quality of life, and surgical expectations (**Table 3**).

### Nominal group

Eleven ASOPRS surgeons were selected to attend the nominal group meeting. All oculoplastic surgeons included performed more than fifty ptosis surgeries annually. Participants were geographically diverse, practicing in the western (4), midwestern (3), eastern (2), and southern (2) regions of the United States.

Preoperative factors were primarily agreed upon by rankings determined in the delphi phase. The nominal group discussion focused on the importance of postoperative factors which were less clearly delineated. The nominal group identified six variables important for the evaluation of ptosis postoperatively: MRD1, presence of lagophthalmos, symmetry of eyelid position, eyelid contour, eyelid crease height/position and ptosis-related quality of life (**Table 4**).

**Table 4:** Factors identified during nominal group discussion as most crucial for postoperative evaluation of ptosis surgery.

Variable	% Respondents	Mean Overall Likert Score
MRD1	87.7%	4.55
Lagophthalmos	36.2%	4.13
Symmetry of eyelid position	22.1%	3.85
Lid contour/lid curve	6.1%	3.71
Lid crease height/position	46.0%	3.56
Ptosis-related quality of life	20.2%	3.39
a. Visual Impairment	40.5%	3.13
b. Dry eye syndrome	35.6%	3.94
c. Comfort	N/A	N/A

The final critical factors identified by this two-stage Delphi and nominal group analysis are shown in **Table 5**. The variables were separated by importance to the preoperative and postoperative periods and categorized by different aspects of the surgical process including, causes of ptosis, surgical planning, anatomy and symmetry, and ptosis-related quality of life.

**Table 5:** Categorical groupings of critical preoperative and postoperative factors in the evaluation of ptosis.

Preoperative	Postoperative
**Causes of Ptosis** 1. Known secondary cause 2. Congenital vs acquired 3. Eyelid surgery or trauma 4. Proptosis / orbital asymmetry 5. Ocular motility	
**Surgical Planning** 1. Levator function 2. Orbicularis/CN VII function 3. Hering’s positivity 4. Anticoagulation 5. Prior history of ptosis surgery	
**Anatomy and Symmetry** 1. MRD1 2. Symmetry of eyelid position 3. Lid contour / lid curve 4. Lid crease height / position 5. Lagophthalmos	**Anatomy and Symmetry** 1. MRD1 2. Symmetry of eyelid position 3. Lid contour / lid curve 4. Lid crease height / position 5. Lagophthalmos
**Ptosis-Related Quality of Life** 1. Visual impairment 2. Dry eye symptoms 3. Comfort	**Ptosis-Related Quality of Life** 1. Visual impairment 2. Dry eye symptoms 3. Comfort

Items in bold are the categorical groupings of critical preoperative and postoperative factors in the evaluation of ptosis.

## Discussion

This two-stage Delphi and nominal group analysis identified factors that are important to oculoplastic surgeons in the preoperative and postoperative evaluation of ptosis. Ptosis etiology and surgical planning were surveyed as more pertinent to the preoperative stage. Anatomy, symmetry and ptosis-related quality of life were applicable outcome measures both before and after surgery.

Many of these measures are readily used by oculoplastic surgeons to evaluate ptosis and surgery efficacy, however there is lack of standardization in the evaluation and reporting of these outcome measures in the literature. The preoperative factors developed through the Delphi technique achieved group consensus easily during the nominal group discussion, in contrast, the postoperative factors were more controversial and required in depth discussion to reach consensus. The preoperative factors fell into two major domains: Surgical Planning (levator function, Herring’s positivity, anticoagulation), and Causes of Ptosis that may alert a surgeon to a non-involutional etiology (known secondary causes of ptosis, proptosis/orbital asymmetry, ocular motility).

Two domains were identified as imperative to both the preoperative and postoperative assessment: Anatomy/Symmetry (MRD1, lid crease symmetry, contour, and symmetry of the eyelid position) and Ptosis-Related Quality of Life (QoL) (dry eye symptoms, visual impairment, and comfort). Developing less quantitative measurements such as Ptosis-Related QoL can be challenging. The nominal group emphasized that dry eye, visual impairment, and comfort were important proxies for the extent to which a patient’s QoL is impaired. These factors ideally would be evaluated pre- and postoperatively to identify improvement with surgery.

However, even for more empirically derived variables there is often little consensus on assessment and quantification. For example, eyelid symmetry has been evaluated using subjective grading (20–23), Image J software (24, 25), and measurements between the lid margin and the pupillary center at the level of the pupil, nasal limbus, and temporal limbus (26). Further, Gordon et al. suggest that small asymmetries in MRD1 may not be apparent to the lay observer, presenting some question as to the appropriate value for what is considered an asymmetric outcome (27).

The effects of ptosis surgery on QoL have previously been measured using scales developed within other specialties and/or without considerable patient input (28–31), a limitation present in this study as well. Further work should be done to solicit patient input on factors they feel have been important throughout their ptosis correction process to determine if there is overlap between the patient and surgeon’s perspective on important outcome measures. Additional work is needed to standardize how each outcome measure is quantified. Using the data presented here, future research can be done to propose a scale for the measurement of important pre- and post-operative factors in the evaluation of ptosis.

## Conclusion

This study distilled the knowledge and experience of a cohort of ASOPRS oculoplastic surgeons to offer consensus on the critical parameters for the preoperative and postoperative evaluation of the patient with ptosis. A standardized scale for ptosis evaluation might have utility in the comparison of surgical selection and outcomes of different surgical techniques. These data may also be useful to better guide surgeons to achieve maximal functional and aesthetic satisfaction for their patients. The preoperative and post operative variables identified in this study might be utilized to develop a standardized scale for the evaluation of ptosis. Future research comparing surgeon-identified success to patient-identified success and reliable assessment of ptosis-related QoL is needed.

## Data Availability

The datasets presented in this article are not readily available because data is not maintained in a publicly accessible repository. Requests to access the datasets should be directed to Daniel Rootman, MD rootman@jsei.ucla.edu.

## References

[1] Sridharan GV Tallis RC Leatherbarrow B Forman WM. A community survey of ptosis of the eyelid and pupil size of elderly people. Age Ageing. (1995) 24:21–4. doi: 10.1093/AGEING/24.1.21, PMID: 7762457

[2] Part B National Summary Data File (Previously known as BESS) | CMS . Available online at: https://www.cms.gov/data-research/statistics-trends-and-reports/part-b-national-summary-data-file (Accessed November 17, 2024).

[3] Allard F Durairaj V. Current techniques in surgical correction of congenital ptosis. Middle East Afr J Ophthalmol. (2010) 17:129. doi: 10.4103/0974-9233.63073, PMID: 20616918 PMC2892127

[4] Ng J Hauck M. Ptosis repair. Facial Plast Surg. (2013) 29:22–5. doi: 10.1055/S-0033-1333831, PMID: 23426748

[5] Cates CA Tyers AG. Outcomes of anterior levator resection in congenital blepharoptosis. Eye (Lond). (2001) 15:770–3. doi: 10.1038/EYE.2001.247, PMID: 11827000

[6] Chang S Lehrman C Itani K Rohrich RJ. A systematic review of comparison of upper eyelid involutional ptosis repair techniques: efficacy and complication rates. Plast Reconstr Surg. (2012) 129:149–57. doi: 10.1097/PRS.0B013E318230A1C7, PMID: 22186506

[7] Meltzer MA Elahi E Taupeka P Flores E. A simplified technique of ptosis repair using a single adjustable suture. Ophthalmology. (2001) 108:1889–92. doi: 10.1016/S0161-6420(01)00712-6, PMID: 11581067

[8] McCulley TJ Kersten RC Kulwin DR Feuer WJ. Outcome and influencing factors of external levator palpebrae superioris aponeurosis advancement for blepharoptosis. Ophthalmic Plast Reconstr Surg. (2003) 19:388–93. doi: 10.1097/01.IOP.0000087071.78407.9A, PMID: 14506424

[9] Whitehouse GM Grigg JRB Martin FJ. Congenital ptosis: results of surgical management. Aust N Z J Ophthalmol. (1995) 23:309–14. doi: 10.1111/J.1442-9071.1995.TB00181.X, PMID: 11980077

[10] Finsterer J. Ptosis: causes, presentation, and management. Aesthetic Plast Surg. (2003) 27:193–204. doi: 10.1007/s00266-003-0127-5, PMID: 12925861

[11] Simon GJB Lee S Schwarcz RM McCann JD Goldberg RA. External levator advancement vs Müller’s muscle-conjunctival resection for correction of upper eyelid involutional ptosis. Am J Ophthalmol. (2005) 140:426–32. doi: 10.1016/J.AJO.2005.03.033, PMID: 16083839

[12] Yoon JS Lee SY. Long-term functional and cosmetic outcomes after frontalis suspension using autogenous fascia lata for pediatric congenital ptosis. Ophthalmology. (2009) 116:1405–14. doi: 10.1016/J.OPHTHA.2009.01.040, PMID: 19481809

[13] Skaat A Fabian ID Spierer A Rosen N Rosner M Ben Simon GJ. Congenital ptosis repair-surgical, cosmetic, and functional outcome: a report of 162 cases. Can J Ophthalmol. (2013) 48:93–8. doi: 10.1016/J.JCJO.2012.09.010, PMID: 23561601

[14] Taherian K Atkinson PL Shekarchian M Scally AJ. Comparative study of the subjective and objective grading of ptosis surgery outcomes. Eye. (2007) 21:5. doi: 10.1038/sj.eye.6702296, PMID: 16498435

[15] Thangaratinam S Redman CW. The delphi technique. Obstetrician Gynaecologist. (2005) 7:120–5. doi: 10.1576/TOAG.7.2.120.27071

[16] Rosowsky E Young AS Malloy MC Van Alphen SPJ Ellison JM. A cross-validation Delphi method approach to the diagnosis and treatment of personality disorders in older adults. Aging Ment Health. (2018) 22:371–8. doi: 10.1080/13607863.2016.1261796, PMID: 27960533

[17] Rowe G Wright G. Expert opinions in forecasting: the role of the delphi technique. Springer Nature Link (2001) 125–44. doi: 10.1007/978-0-306-47630-3_7

[18] CDC. Gaining consensus among stakeholders through the nominal group technique (2018). Available online at: https://www.cdc.gov/healthyyouth/evaluation/pdf/brief7.pdf (Accessed November 17, 2024).

[19] Allen J Dyas J Jones M. Building consensus in health care: a guide to using the nominal group technique. Br J Community Nurs. (2004) 9:110–4. doi: 10.12968/BJCN.2004.9.3.12432, PMID: 15028996

[20] Goldberg RA Lew H. Cosmetic outcome of posterior approach ptosis surgery (An American ophthalmological society thesis). Trans Am Ophthalmol Soc. (2011) 109:157., PMID: 22253486 PMC3259674

[21] Matsuda H Sakai T Takahashi Y Nakano T. Surgical outcomes of the anterior versus posterior approach for advancement of the levator aponeurosis in Japanese patients. J Plast Reconstr Aesthet Surg. (2020) 73:2001–9. doi: 10.1016/J.BJPS.2020.08.051, PMID: 32912723

[22] Frueh BR Musch DC McDonald HMB. Efficacy and efficiency of a small-incision, minimal dissection procedure versus a traditional approach for correcting aponeurotic ptosis. Ophthalmology. (2004) 111:2158–63. doi: 10.1016/J.OPHTHA.2004.07.019, PMID: 15582068

[23] Papageorgiou KI Ang M Chang SH Kohn J Martinez S Goldberg RA. Aesthetic considerations in upper eyelid retraction surgery. Ophthalmic Plast Reconstr Surg. (2012) 28:419–23. doi: 10.1097/IOP.0B013E318263C56E, PMID: 23138201

[24] Young W Scofield-Kaplan SM Levy RE Keenum Z Mancini R. Change in lower eyelid contour following ectropion repair with lateral tarsal strip. Ophthalmic Plast Reconstr Surg. (2020) 36:557–61. doi: 10.1097/IOP.0000000000001634, PMID: 32205778

[25] Golbert MB Garcia DM Akaishi PMS e Cruz AAV. Upper eyelid contour symmetry measurement with Bézier curves. Arq Bras Oftalmol. (2020) 83:28–32. doi: 10.5935/0004-2749.20200002, PMID: 31531548 PMC11984440

[26] Sendul SY Atilgan CU Dirim B Yildiz AM Arslan GD Demir ST. The effect of two different frontalis sling approaches on postoperative eyelid contour: A comparative study. Aesthetic Plast Surg. (2020) 44:381–9. doi: 10.1007/S00266-019-01574-4, PMID: 31844944

[27] Gordon MJ Christenbury JG Bokman CL Rootman DB Goldberg RA. Ptosis sensitivity threshold for the lay observer. Ann Plast Surg. (2018) 81:364–6. doi: 10.1097/SAP.0000000000001524, PMID: 29905610

[28] Federici TJ Meyer DR Lininger LL. Correlation of the vision-related functional impairment associated with blepharoptosis and the impact of blepharoptosis surgery. Ophthalmology. (1999) 106:1705–12. doi: 10.1016/S0161-6420(99)90354-8, PMID: 10485538

[29] Battu VK Meyer DR Wobig JL. Improvement in subjective visual function and quality of life outcome measures after blepharoptosis surgery. Am J Ophthalmol. (1996) 121:677–86. doi: 10.1016/S0002-9394(14)70634-8, PMID: 8644811

[30] Mahroo OA Hysi PG Dey S Gavin EA Hammond CJ Jones CA. Outcomes of ptosis surgery assessed using a patient-reported outcome measure: an exploration of time effects. Br J Ophthalmol. (2014) 98:387–90. doi: 10.1136/BJOPHTHALMOL-2013-303946, PMID: 24344234

[31] Smith HB Jyothi SB Mahroo OAR Shams PN Sira M Dey T. Patient-reported benefit from oculoplastic surgery. Eye (Lond). (2012) 26:1418–23. doi: 10.1038/EYE.2012.188, PMID: 22975655 PMC3496101

